# Interplay of replication timing, DNA repair, and translesion synthesis in UV mutagenesis in yeast

**DOI:** 10.1080/19491034.2025.2476935

**Published:** 2025-03-13

**Authors:** Allysa Sewell, John J. Wyrick

**Affiliations:** School of Molecular Biosciences, Biotechnology Life Sciences, Washington State University, Pullman, WA, USA

**Keywords:** Complex mutations, DNA damage, global genomic-nucleotide excision repair, nucleotide excision repair, ultraviolet light

## Abstract

Replication timing during S-phase impacts mutation rates in yeast and human cancers; however, the exact mechanism involved remains unclear. Here, we analyze the impact of replication timing on UV mutagenesis in *Saccharomyces cerevisiae*. Our analysis indicates that UV mutations are enriched in early-replicating regions of the genome in wild-type cells, but in cells deficient in global genomic-nucleotide excision repair (GG-NER), mutations are enriched in late-replicating regions. Analysis of UV damage maps revealed that cyclobutane pyrimidine dimers are enriched in late-replicating regions, but this enrichment is almost entirely due to repetitive ribosomal DNA. Complex mutations typically associated with TLS activity are also elevated in late-replicating regions in GG-NER deficient cells. We propose that UV mutagenesis is higher in early-replicating regions in repair-competent cells because there is less time to repair the lesion prior to undergoing replication. However, in the absence of GG-NER, increased TLS activity promotes UV mutagenesis in late-replicating regions.

## Introduction

Exposure to ultraviolet (UV) light is a primary risk factor for skin carcinogenesis because UV radiation induces damage in DNA that causes mutations during replication. The major class of UV-induced DNA damage is cyclobutane pyrimidine dimers (CPDs), which form between adjacent pyrimidine bases (i.e., TT, TC, CT, or CC) [1–3]. If not efficiently repaired by the nucleotide excision repair (NER) pathway [4–7], these helix-distorting lesions can block ongoing DNA replication [2,8]. Replicative bypass of CPDs and other helix-distorting UV photoproducts is thought to involve either an error-free recombination-based mechanism, such as template switching, or bypass by translesion DNA synthesis (TLS) [9,10]. This latter mechanism involves potentially error-prone DNA synthesis past the lesion by TLS DNA polymerases, which is likely responsible for most UV-induced mutations [9,11].

Multiple studies suggest that UV-induced mutations and spontaneous mutation classes do not accumulate at a uniform rate during S-phase [12–15]. Genome sequencing of human skin cancers such as melanoma has revealed that a larger fraction of somatic mutations occurs in late-replicating regions of the genome relative to earlier-replicating regions [12,16,17]. Elevated mutation rates in late-replicating regions of the genome have been observed not only in many other cancer types (e.g., [12,18,19]), but also in human germline mutations [15]. Multiple mechanisms have been proposed to explain this correlation, including differences in the deoxynucleotide pool during S-phase, in the utilization of TLS polymerases to bypass lesions, and in DNA repair activity due to regional differences in chromatin accessibility [13,15–17,19,20]. For example, the relative accessibility of UV damage to repair by the global genomic-NER (GG-NER) pathway significantly contributes to replication timing-associated differences in mutation frequency in human skin cancers [16,17]. Despite intense study, to what extent these and potentially other mechanisms contribute to elevated mutation rates in late-replicating regions of the genome is still largely unclear. Moreover, a few mutational processes (e.g., mutations induced by APOBEC cytidine deaminases) are enriched in early-replicating regions of the genome [21,22], but the mechanism responsible for this association is unknown.

Previous research has also indicated that replication timing impacts mutation frequencies in yeast (*S. cerevisiae*), with rates being elevated in late-replicating regions [13,14,23]. For example, one study measured the spontaneous mutation rates in a *URA3* reporter gene integrated at 43 different locations in yeast and found a six-fold variation in *URA3* mutation frequency across these 43 locations [13]. This disparity could largely be explained by the replication timing of the genomic region in which the reporter gene was integrated, based on a previously published genome-wide map of replication timing [24], with locations in late-replicating regions having significantly higher mutation frequencies. However, other studies reported a much smaller variation in mutation frequencies with replication timing in yeast, with spontaneous mutation rates showing only a 20–30% increase in late-replicating regions [14,23]. While these studies examined spontaneous mutation rates, the effect of replication timing on the frequency of mutations induced by exogenous damaging agents such as UV light in yeast is unknown. The temporal regulation of error-prone TLS activity during S-phase has been postulated as a potential cause of the increase in spontaneous mutation frequency during late replication observed in yeast cells [13], since the *REV1* TLS polymerase is expressed exclusively during late S-phase and G2/M-phases of the cell cycle [25]. However, whether a similar mechanism regulates UV-induced mutation frequency in yeast is unclear.

We have previously used genome sequencing to identify UV-induced mutations in repair-proficient and repair-deficient cells repeatedly exposed to either UVC or UVB light [26,27]. Here, we use this compendium of more than 60,000 UV-induced mutations to characterize how replication timing in yeast affects UV mutagenesis.

## Materials and methods

### Data sources and timing map

Analyses were performed for data from WT, *rad16*Δ, and *rad26Δ* yeast strains exposed to either UVC or UVB radiation. The mutation data are from previously performed whole-genome sequencing of yeast cells subjected to UV passaging experiments [26,27] and are available on NCBI Sequence Read Archive (SRA; https://www.ncbi.nlm.nih.gov/sra) under BioProject accession number SRA: PRJNA605561 (UVC) and PRJNA888347 (UVB). Single-base substitutions and complex mutations were extracted from these data sets and put into BED files. CPD-seq data were obtained from published UV damage and repair experiments in yeast [28,29] and can be obtained from the Gene Expression Omnibus (GEO) database using accessions: GSE79977 (WT) and GSE131101 (*rad16*∆). CPD-seq wig files were converted to BED files and analyzed in a similar manner as the mutation data. A CPD count was assigned to both positions in the lesion-forming dipyrimidine. Dipyrimidine sequence counts were obtained from FASTA files derived from the identified early, middle, and late-replicating regions of the genome. Analysis of the CPD-seq data and mutation data was also repeated excluding in CPDs and mutations in rDNA repeats associated with the following genome coordinates: chrXII:451430–468920 and chrXII:489928–490455.

The replication timing map was derived from data lifted to the Saccer3 genome assembly from Raghuraman et al. [24], which provides replication time values by genome location at 500 base-pair intervals.

### Assigning replication times

A custom Python script was used to classify mutations from our previously obtained whole-genome sequencing data sets from UV passaging experiments based on when they occur during S-phase. The code is available on GitHub in the repository allysasewell/DNA_replication timing. Using the timing map from Raghuraman et al. [24], approximate replication times were assigned to each single-base substitution in the inputted BED files. This was accomplished by identifying the interval each mutation from the BED file falls within and using the following formula to calculate replication time:

time = time1 + (time2−time1)*[(pos−pos1)/(pos2−pos1)]

In this formula, pos1 is the first position in the interval from the timing map that is replicated at time1, pos2 is the second position in the interval replicated at time2, and pos is the position of the mutation in the BED file. Mutations in regions not covered in the intervals from the timing map, such as the first approximately 250 base pairs of each chromosome, were excluded.

### Data classification and analysis

Once replication times were assigned, substitution mutations could be categorized as early, middle, or late. Early, middle, and late-replicating regions were defined for the *S. cerevisiae* genome by sorting data from the Raghuraman et al. replication timing map [24] and dividing it into three bins each encompassing a third of the genome. This resulted in times from 0 to 27.67 minutes being classified as early, 27.67–34.28 min as middle, and times after 34.28 min as late. Counts were obtained for each replication timing bin and compared with expected values calculated based on trinucleotide counts for each bin, as determined by inputting FASTA files for each chromosome and assigning a replication time for the middle base of each trinucleotide as described above, and overall mutation frequencies for each trinucleotide sequence context. Additionally, each bin was further subdivided into three more bins for a total of nine timepoints for use in linear regression analysis relating mutation frequency for each bin to its average replication time.

Analysis of mutations and CPDs associated with different replication timing categories and individual chromosomes was performed similarly as described above.

### Analyzing complex mutations

To identify complex mutations, each yeast isolate was considered separately, and if a mutation was within 10 base pairs of the preceding or subsequent mutation, it was added to the list of complex mutations, if not already present. Mutations could be substitutions, insertions, or deletions. BED files were created with all the complex mutations, and replication timing was analyzed as described above. When calculating expected values for complex mutations, it was assumed that the proportions of mutations in early, middle, and late categories would be identical to those observed for single-base substitutions (SBS). Mutation spectra for complex mutations included only SBS occurring in complex mutation events.

A custom Python script was used to find the cosine similarities between the mutation spectra for complex mutations and each of the mutation signatures listed in the COSMIC database [30]. The lists of mutation frequencies in each trinucleotide context were treated as vectors, so the dot product of the complex mutation frequencies and the COSMIC mutation signature frequencies was calculated and divided by the product of the vector magnitudes.

## Results

To explore the impact of replication timing during S-phase on UV mutagenesis in yeast, we analyzed a compendium of ~12,000 UV-induced single base substitutions (SBS). These mutations were identified by whole-genome sequencing of wild-type (WT) yeast isolates exposed to 15 doses of 25 J/m^2^ of UVC light [26]. The replication timing of each mutation was estimated from a published whole-genome map of replication timing in yeast [24]. Using this replication timing map, we divided the genome into early, middle, and late-replicating segments, each containing approximately equivalent amounts of genomic sequence (see Methods). We used this analytical framework to compare the total number of UV-induced mutations in early, middle, and late-replicating regions of the yeast genome.

**Figure 1.**
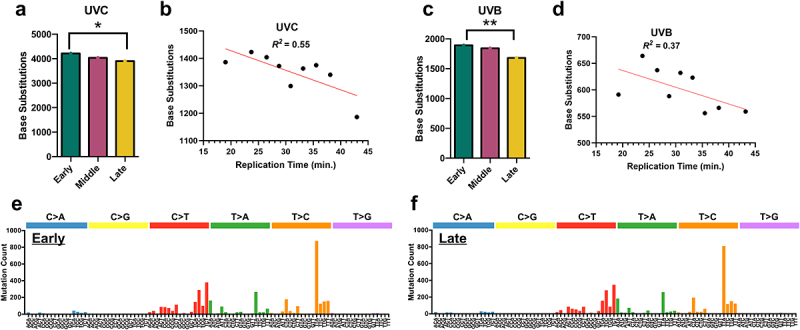
UV-induced single base substitutions in wild-type (WT) cells are elevated in early-replicating regions of the yeast genome. (a) The number of single-base substitutions in early, middle, and late-replicating regions of the genome for UVC-treated wild-type (WT) yeast. Mutations are derived from genome-wide sequencing of WT yeast exposed to 15 doses of UVC light (25 J/m^2^ per dose), as described in [26]. *p < 0.05 based on chi-square analysis of observed mutation counts relative to the expected mutations counts based on tri-nucleotide sequence contexts of the early, middle, and late-replicating genomic sequences. (b) Linear regression results plotting number of single-base substitution mutations by replication time for UVC-exposed WT yeast. The regression equation is y = −7.125× + 1570 with a Pearson’s R of − 0.74 and a p-value of 0.0216. Simple linear regression was performed using GraphPad prism version 10.0.3. (c) The number of single-base substitutions in early, middle, and late-replicating regions of the genome for UVB-treated wild-type (WT) yeast. Mutations are derived from genome-wide sequencing of WT yeast exposed to 15 doses of UVB light (300 J/m^2^ per dose), as described in [27]. **p < 0.005 based on chi-square analysis. (d) Linear regression results plotting number of single-base substitution mutations by replication time for UVB-exposed wild-type yeast. The regression equation is y = −3.158× + 699.7 with a Pearson’s R of − 0.61 and a p-value of 0.0841. (e) Mutation spectrum for single-base substitutions (C>a, C>G, C>T, T>A, T>C, or T>G) in early S-phase for WT UVC-exposed yeast cells. Counts for all trinucleotide contexts are included, with the mutated base in the middle. (f) Mutation spectrum for single-base substitutions in late S-phase for WT UVC-exposed yeast cells.

This analysis indicated that in WT yeast the number of UV-induced mutations was ~8% higher in early-replicating regions of the genome relative to late-replicating regions (Figure 1a). Genomic regions with middle replication timing had an intermediate count of mutations, ~3% higher than late-replicating regions. As a control, we estimated the expected number of mutations in each region due to DNA sequence composition alone, which were calculated from the underlying DNA sequence using observed mutation frequencies for each trinucleotide sequence context (see Methods). These expected mutation counts showed relatively little variation (~1% difference between categories), and statistical analysis indicated that the observed mutation counts in the replication timing categories significantly differed from the expected count (*p* = 0.019). To further characterize the dependence of UV mutagenesis on replication timing, we subdivided the early, middle, and late-replicating regions into nine total bins ordered by replication timing and quantified the number of mutations in each bin. Linear regression analysis revealed a significant negative correlation between mutation count and replication timing (Figure 1b, Pearson’s *R* = −0.74, *p* = 0.0216), consistent with the hypothesis that the accumulation of UV mutations is elevated early in S-phase.

To further test this hypothesis, we analyzed the association between replication timing and UV mutagenesis in a separate compendium of >5000 single base substitutions derived from whole-genome sequencing of WT yeast exposed to 15 doses of 300 J/m^2^ of UVB light [27]. UVB and UVC light generate similar classes of UV photoproducts in DNA and a similar spectrum of mutations, albeit at somewhat different proportions [26,27,31]. Our analysis indicated that UVB-induced mutations in WT cells are elevated in early-replicating regions of the yeast genome, with ~13% more mutations than in late-replicating regions (Figure 1c). In contrast, the expected mutation counts based on sequence composition alone only varied by <1% between each replication timing category and were significantly different than the observed mutation counts (*p* = 0.0035). Linear regression analysis of UVB-induced mutations indicated a low-confidence negative correlation between replication timing and mutation accumulation (Figure 1d, Pearson’s *R* = −0.61), as the correlation was not statistically significant (*p* = 0.084), likely because of the smaller number of UVB-induced mutations analyzed.

We also compared the trinucleotide spectra of UV-induced mutations in early and late-replicating regions of the yeast genome. Analysis of UVC-induced mutations indicated a very similar mutation spectrum in early and late-replicating regions of the genome (Figure 1e,f). In support of this finding, quantitative analysis of the frequency of each mutation class in different trinucleotide sequence contexts (e.g., TCT>TTT, etc.) revealed a very high similarity in the mutation spectra of the early and late-replicating regions of the genome (R^2^ = 0.99, Supplemental Figure 1a). Comparisons with the mutation spectrum of middle replication timing regions also showed very high similarity (Supplemental Figure 1b,c). We observed similar results for the UVB mutation data (Supplemental Figure 1d-f). Taken together, these findings indicate that UV-induced mutations in repair-proficient WT yeast are elevated in early-replicating regions of the genome, but the spectrum of mutations is not significantly altered by replication timing.

### Apparent elevation of UV damage in late-replicating regions is due to ribosomal DNA repeats

We wondered whether differences in UV damage formation or repair efficiency might explain the observed variation in mutation counts in early versus late-replicating regions of the yeast genome. To test this hypothesis, we analyzed published yeast CPD-seq data, which maps UV-induced CPD lesions at single-nucleotide resolution across the yeast genome [29]. We initially examined UV-induced CPD formation by assigning lesions identified by CPD-seq to early, middle, or late-replicating regions of the genome (see Methods).

**Figure 2.**
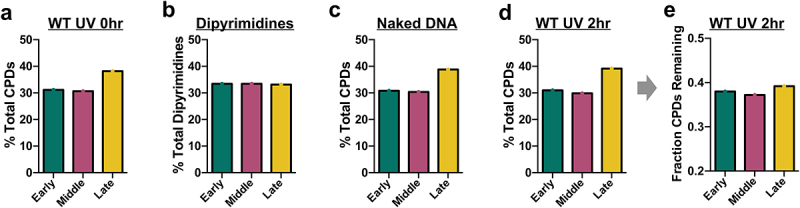
Analysis of UV-induced CPD formation and repair in early, middle, and late-replicating regions of the yeast genome. (a) Percentage of CPDs in early, middle, and late-replicating regions of the yeast genome immediately after UVC-radiation of WT cells (0 h). CPD counts were determined from published cpd-seq data [29]. (b) Percentage of dipyrimidine sequences in early, middle, and late-replicating regions of the yeast genome. (c) Same as panel a, except for UVC-radiation (90 J/m2) of isolated yeast genomic DNA. (d) Same as panel a, except after 2 h of repair in UV-radiated WT cells (2 h). (e) Fraction of CPDs remaining after 2 h of repair in UV-radiated WT cells relative to the 0 h control in early, middle, and late-replicating regions of the yeast genome.

Our results indicated that late-replicating regions of the genome are enriched for CPD lesions, containing ~20–25% more damage than early or middle-replicating regions (Figure 2a and Supplemental Figure S2a). One possible explanation of this result is that late-replicating regions could have a higher frequency of CPD-forming dipyrimidine sequences. However, sequence analysis indicated that late-replicating regions had a slightly lower number of dipyrimidine sequences than early or middle-replicating regions (Figure 2b). Analysis of CPD-seq data derived from isolated yeast genomic DNA that was UV irradiated *in vitro* [29] showed a similar pattern of CPD enrichment in late-replicating regions of the genome (Figure 2c and Supplemental Figure S2b), suggesting that the overall DNA sequence composition of late-replicating regions, and not DNA binding by cellular proteins, is primarily responsible for elevated UV damage formation. Analysis of CPD-seq data in WT cells following 2 h of repair [29] showed a similar degree of enrichment of CPDs in late-replicating regions relative to early or middle-replicating regions (i.e., ~25–30% higher CPDs in late-replicating regions, Figure 2d). Analysis of the fraction of CPDs remaining following 2 h repair in WT cells relative to the 0 h control revealed a roughly similar fraction of unrepaired CPDs in each of the replication timing categories (Figure 2e). In summary, our data indicate that NER efficiency is roughly similar in late, middle, and early-replicating genomic regions in WT cells, while UV damage is specifically elevated in late-replicating regions.

**Figure 3.**
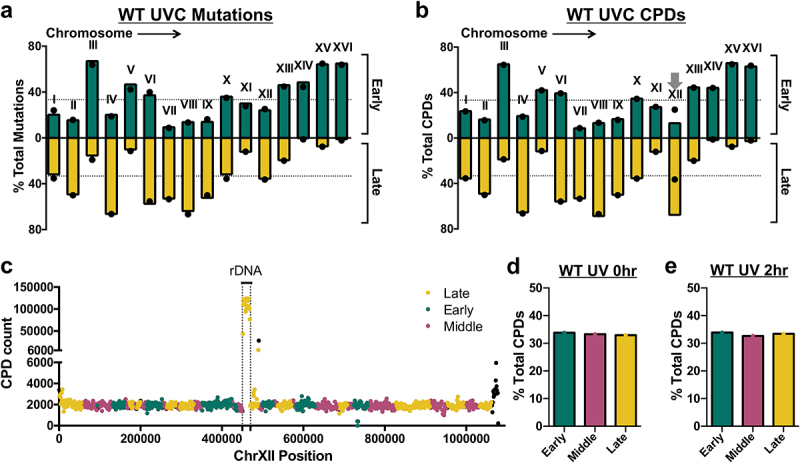
Comparison of the percentages of mutations and CPDs incurred in early and late-replicating regions in UVC-exposed wild-type (WT) yeast for individual chromosomes. (a) Mutation percentages in early and late-replicating DNA (green and yellow bars, respectively) were derived from whole-genome sequencing of UVC-exposed yeast cells [26] and plotted for each yeast chromosome. Expected values (black dots) were calculated based on the DNA sequence context using trinucleotide counts by region (early, middle, or late-replicating) and overall mutation frequencies for each trinucleotide context. Each individual chromosome is labeled. The dotted lines represent the expected proportion of mutations (i.e., 33% or 1/3^rd^) for early and late-replicating regions across the genome as a whole. (b) Same as panel a, but showing percentages of CPDs in early and late-replicating DNA derived from cpd-seq data from UV-radiated WT cells (0 h; data from [29]). Black circles indicate the percentages of dipyrimidine sequences in early and late-replicating DNA for each chromosome. Gray arrow indicates a significant discrepancy between percentage of total CPDs and dipyrimidine sequences in chromosome XII. (c) Plot of CPD counts for 1kb bins spanning chromosome XII. Each band is indicated by a single circle, which is color-coded by whether it occurs in a late, early, or middle-replicating region of the chromosome. Black circles indicate regions in which the entire bin does not fall into a single replication timing category, or where replication timing data was not available. The ribosomal DNA (rDNA) locus is indicated on the plot. (d-e) Percentage of total CPDs in each replication timing category for UV-radiated WT cells, either (d) immediately after UV-radiation or (e) after 2 h repair, after removing CPDs occurring in repetitive rDNA locus (see materials and methods).

We wondered whether the enrichment of UV-induced mutations in early replicating regions and CPDs in late-replicating regions were consistent across individual chromosomes. To address this question, we initially tested how replication timing impacted UV mutagenesis along individual yeast chromosomes. For UVC-irradiated WT cells, the percentage of mutations in early-replicating regions varied from chromosome to chromosome (Figure 3a). For example, in chromosomes III, XV, and XVI, more than 50% of the mutations were in early-replicating regions, while in chromosomes VII, VIII, and IX, fewer than 15% of mutations were in early-replicating regions and more than 50% of mutations were late-replicating (Figure 3a). Associations with early or late-replicating regions in each chromosome largely matched the expected mutation counts based on DNA sequence composition (see black circles in Figure 3a), indicating that the chromosome-to-chromosome variation observed was largely due to chromosome-specific differences in replication timing. For example, more than 50% of the DNA sequence in chromosomes III, XV, and XVI is early-replicating, which can account for why such a high proportion of mutations are assigned to this replication timing category in these chromosomes. Analysis of UVB-induced mutations in WT yeast showed a similar pattern (Supplemental Figure S3).

In addition to this chromosome-to-chromosome variation, the UVC mutation data showed a general trend of fewer mutations in late-replicating regions than expected by DNA sequence composition across the different chromosomes (Figure 3a). Statistical analysis using a Wilcoxon matched-pairs signed rank test confirmed that the percentage of total mutations in late-replicating regions across the different chromosomes was smaller than expected (*p* = 0.025). This finding is consistent with our previous analysis indicating that, for the genome as a whole, there are fewer mutations in late-replicating regions than early or middle-replicating regions of the genome (see Figure 1).

Similar analysis of CPD lesions in individual chromosomes for UV radiated WT cells (0 h) revealed a comparable pattern of chromosome-to-chromosome variability in the distribution of CPD lesions in early versus late-replicating regions (Figure 3b). Again, this variability could largely be explained by the fraction of early versus late-replicating DNA sequence in each chromosome, as the percentage of CPD-forming dipyrimidine sequences (black circles in Figure 3b) largely mirrored the percentage of CPDs in each chromosome.

One notable exception was chromosome XII, where there was a much higher percentage of CPDs in late-replicating regions than expected based on the percentage of dipyrimidine sequences (Figure 3b and Supplemental Figure S4a). Closer inspection of CPD counts in 1kb bins along chromosome XII revealed a ~ 40-fold enrichment of CPDs associated with a specific region of late-replicating DNA that contains the ribosomal DNA (rDNA) locus (Figure 3c). The rDNA locus in *S. cerevisiae* typically contains 150–200 tandem repeats of a cassette containing the four ribosomal RNA genes [32,33], but only two rDNA repeats are included in the Saccer3 reference genome assembly [34]. This discrepancy can explain why CPDs are strongly enriched at the late-replicating rDNA locus in chromosome XII, and why this enrichment was not accounted for when calculating the distribution of dipyrimidine sequences in early versus late-replicating regions of chromosome XII (Supplemental Figure 4a). Filtering out CPDs resident in the rDNA locus eliminated the apparent enrichment of CPDs in late-replicating regions of the yeast genome (Figure 3d,e and Supplemental Figure S4b-d). Taken together, these findings indicate that the apparent enrichment of CPD lesions in late-replicating regions of the genome is caused by the repetitive nature of the late-replicating rDNA locus in chromosome XII. Instead, this new analysis indicates that CPD levels immediately after UV radiation (0 h), in UV radiated naked DNA, or after 2 h of repair are very similar between early and late-replicating regions of the genome and largely coincide with the frequency of dipyrimidine sequences (Figure 3d,e and Supplemental Figure S4b-d).

To account for the possibility of a similar bias in our mutation data due to the rDNA repeats, which tend to be located in late-replicating regions of chromosome XII, we repeated our analysis of UV mutations excluding these regions. However, the effect of eliminating these regions on replication timing trends was negligible, likely because few mutations were called in the repetitive rDNA locus. After excluding the rDNA locus, we still observed significant enrichment of mutations in early-replicating regions of the yeast genome relative to late-replicating regions (Supplemental Figure S5), confirming our original results.

## *UV mutations are elevated in late-replicating regions in GG-NER-deficient* rad16∆ *cells*

**Figure 4.**
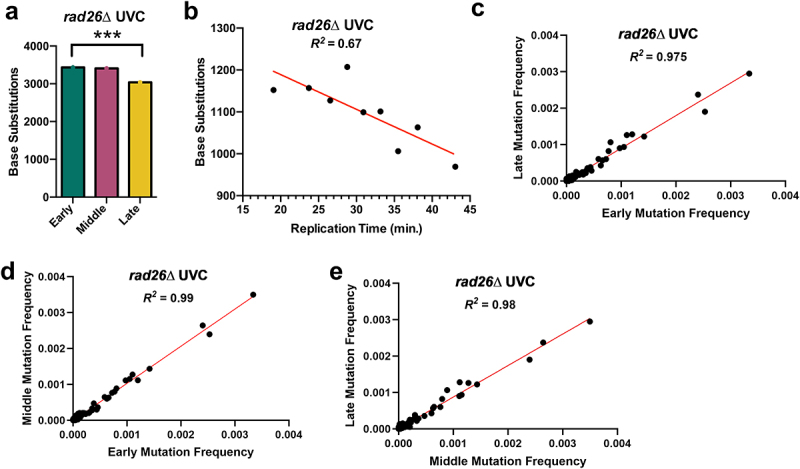
UV-induced single base substitutions in *rad26*∆ cells are elevated in early-replicating regions of the yeast genome. (a) The number of single-base substitutions in early, middle, and late-replicating regions of the genome for UVC-treated *rad26*∆ yeast. Mutations are derived from genome-wide sequencing of WT yeast exposed to 15 doses of UVC light (25 J/m^2^ per dose), as described in [26]. *p < 0.0005 based on chi-square analysis of observed mutation counts relative to the expected mutations counts based on tri-nucleotide sequence context of the early, middle, and late-replicating genomic sequences. (b) Linear regression results plotting number of single-base substitution mutations by replication time for UVC-exposed *rad26*Δ yeast. The regression equation is y = −8.288× + 1355 with a Pearson’s R of − 0.82 and a p-value of 0.0072. Simple linear regression was performed using GraphPad prism version 10.0.3. (c-e) Comparison of mutation spectra for each trinucleotide context at different replication times for *rad26*∆ yeast cells exposed to UVC. Correlation/linear regression analysis was performed using GraphPad prism version 10.0.3. *p* < 0.0005 for each comparison.

Previous studies in human skin cancers have suggested that differential NER activity can potentially explain variations in mutation density associated with replication timing [16,17]. To investigate how NER activity influences replication timing-associated mutation rates in yeast, we analyzed UV-induced mutations derived from yeast strains deficient in either the transcription coupled-nucleotide excision repair (TC-NER) or global genomic-nucleotide excision repair (GG-NER) pathway. First, we analyzed ~10,000 mutations derived from whole-genome sequencing of TC-NER deficient *rad26*∆ cells that had been exposed to 15 doses of 25 J/m^2^ UVC light [26]. This analysis revealed a similar pattern to WT cells, with ~13% more mutations in early-replicating regions of the genome than late-replicating regions (Figure 4a). Again, the observed mutation counts in each replication timing category were significantly different than the expected counts derived from DNA sequence composition alone (*p* < 0.0005). Linear regression analysis also revealed a significant negative correlation between time of replication in S-phase and mutation count (Figure 4b, Pearson’s *R* = −0.82, *p* = 0.0072). The mutation spectra of early, middle, and late-replicating regions were highly similar (Figure 4c-e), consistent with our analysis of UV mutations in WT cells. Taken together, these findings suggest that TC-NER does not contribute to the difference in mutation counts in early- versus late-replicating regions of the genome.

**Figure 5.**
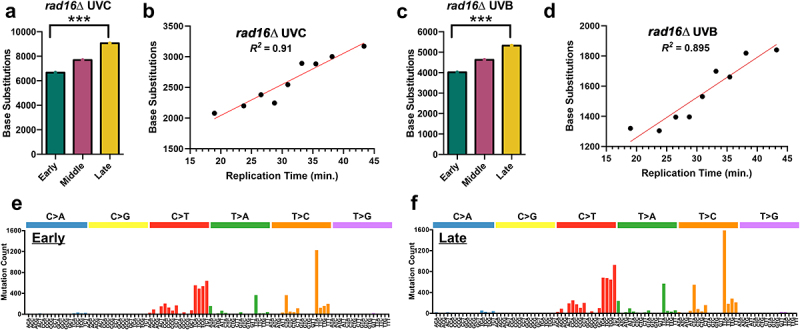
UV-induced single base substitutions in *rad16*∆ cells are elevated in late-replicating regions of the yeast genome. (a) The number of single-base substitutions in early, middle, and late-replicating regions of the genome for UVC-treated *rad16*∆ yeast. Mutations are derived from genome-wide sequencing of *rad16*∆ yeast exposed to 15 doses of UVC light (12.5 J/m^2^ per dose), as described in [26]. *p < 0.0005 based on chi-square analysis of observed mutation counts relative to the expected mutations counts based on tri-nucleotide sequence context of the early, middle, and late-replicating genomic sequences. (b) Linear regression results plotting number of single-base substitution mutations by replication time for UVC-exposed *rad16*Δ yeast. The regression equation is y = 50.67× + 1029 with a Pearson’s R of 0.96 and a p-value of <0.0005. Simple linear regression was performed using GraphPad prism version 10.0.3. (c) The number of single-base substitutions in early, middle, and late-replicating regions of the genome for UVB-treated *rad16*∆ yeast. Mutations are derived from genome-wide sequencing of WT yeast exposed to 15 doses of UVB light (150 J/m^2^ per dose), as described in [27]. **p < 0.0005 based on chi-square analysis. (d) Linear regression results plotting number of single-base substitution mutations by replication time for UVB-exposed *rad16Δ* yeast. The regression equation is y = 26.6× + 727.1, with a Pearson’s R of 0.95 and a p-value <0.0005. (e) Mutation spectrum for single-base substitutions (C>a, C>G, C>T, T>A, T>C, or T>G) in early S-phase for *rad16*∆ UVC-exposed yeast cells. Counts for all trinucleotide contexts are included, with the mutated base in the middle. (f) Mutation spectrum for single-base substitutions in late S-phase for *rad16*∆ UVC-exposed yeast cells.

In contrast, similar analysis of ~23,000 mutations from GG-NER defective *rad16*∆ cells that had been exposed to 15 doses of UVC light (12.5 J/m^2^) [26] showed a very different pattern than WT or *rad26*∆ cells. In *rad16*∆ cells, UVC-induced mutations were significantly elevated in late-replicating regions of the genome, with ~36% more mutations than in early-replicating DNA (Figure 5a). Consistently, the observed mutation counts in early, middle, and late-replicating regions of the genome significantly differed from the expected mutation counts based on DNA sequence composition (*p* < 0.0005), even if the rDNA locus is excluded (Supplemental Figure S6). Moreover, regression analysis also indicated a positive linear relationship between replication time and mutation count (Figure 5b, Pearson’s *R* = 0.96, *p* < 0.0005). Very similar results were obtained when we analyzed a separate cohort of ~14,000 mutations derived from whole-genome sequencing of *rad16*∆ cells that have been irradiated 15 times with 150 J/m^2^ of UVB (Figure 5c,d) [27]. In UVB-irradiated *rad16*∆ cells, late-replicating regions had a ~ 32% increase in mutations relative to early-replicating regions of the genome (Figure 5c), and the observed counts of mutations in each replication timing category significantly differed from the expected count based on DNA sequence composition (*p* < 0.0005). Regression analysis also indicated that mutation counts showed a significant positive correlation with replication timing (Figure 5d, Pearson’s *R* = 0.95, *p* < 0.0005).

While UV-induced mutation frequency in *rad16*∆ was elevated in late-replicating regions, we did not observe any significant differences in the mutation spectrum in genomic regions with different replication timing. For example, in UVC-irradiated *rad16*∆ cells, the trinucleotide mutation spectrum of early-replicating regions was very similar to that of late-replicating regions (Figure 5e,f), having an *R*^*2*^ of 0.99 (Supplemental Figure S7a). Middle-replicating regions also had a very similar mutation spectrum as early and late-replicating regions (Supplemental Figure S7b,c). Similar results were obtained when analyzing the mutation spectra of mutations arising in UVB-irradiated *rad16*∆ cells (Supplemental Figure S7d-f).

**Figure 6.**
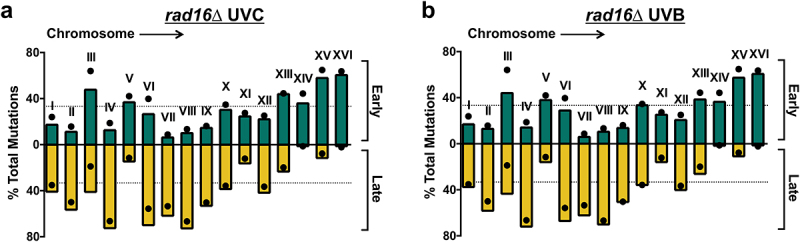
Analysis of UV mutations in *rad16*∆ cells for individual yeast chromosomes. (a-b) Mutation percentages in early- and late-replicating DNA (green and yellow bars, respectively) were derived from whole-genome sequencing of (a) UVC-exposed and (b) UVB-exposed *rad16*∆ yeast cells [26] and plotted for each yeast chromosome. Expected values (black dots) were calculated based on the DNA sequence context using trinucleotide counts by region (early, middle, or late-replicating) and overall mutation frequencies for each trinucleotide context. The dotted lines represent the expected proportion of mutations (i.e., 33% or 1/3^rd^) for early and late-replicating regions across the genome as a whole. Each individual chromosome is labeled.

Analysis of the distribution of mutations in UVC-irradiated *rad16*∆ cells across different chromosomes revealed a chromosome-to-chromosome variation similar to that observed in WT cells (compare Figures 3a and 6a). However, there was a general trend of more mutations in late-replicating regions relative to the expected percentage based on sequence composition (Figure 6a). Very similar patterns were observed when we analyzed a separate set of UVB-induced mutations in *rad16*∆ cells (Figure 6b). Statistical analysis using the Wilcoxon matched-pairs signed rank test validated that the count of *rad16*∆ UVC-induced mutations in late-replicating regions across different chromosomes was higher than expected (*p* = 0.0002). Taken together, these findings indicate that in GG-NER -deficient *rad16*∆ cells, UV-induced mutations are significantly elevated in late-replicating regions, but the corresponding mutation spectrum is unaffected by replication timing.

### Complex mutations are elevated in late-replicating regions in rad16∆ cells

**Figure 7.**
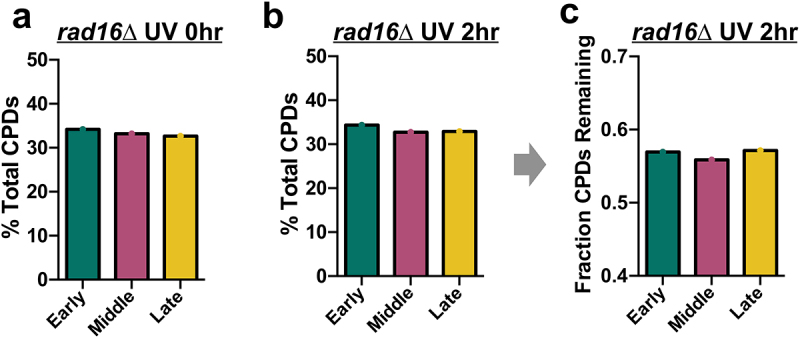
Analysis of UV-induced CPD formation and repair in GG-NER deficient *rad16*∆ cells in early, middle, and late-replicating regions of the yeast genome. (a) Percentage of CPDs in early, middle, and late-replicating regions of the yeast genome immediately after UVC-radiation of *rad16*∆ cells (0 h). CPD counts determined from published cpd-seq data [28], after removing CPDs associated with ribosomal DNA (rDNA) repeats. (b) Same as panel a, except after 2 h of repair in UV-radiated *rad16*∆ cells (2 h). (c) Fraction of CPDs remaining after 2 h of repair in UV-radiated *rad16*∆ cells relative to the 0 h control in early, middle, and late-replicating regions of the yeast genome, after removing CPDs associated with ribosomal DNA (rDNA) repeats.

To investigate the potential causes of elevated mutation counts in late-replicating regions, we analyzed published CPD-seq data from *rad16*∆ cells [28]. After excluding repetitive rDNA regions (see Figure 3), our analysis indicated that initial CPD formation in *rad16*∆ cells is roughly equivalent in late-replicating regions of the genome relative to middle or early-replicating regions (Figure 7a and Supplemental Figure S8a). Similar results were obtained after 2 h of repair (Figure 7b and Supplemental Figure S8b). Analysis of the fraction of CPDs remaining following 2 h repair in *rad16*∆ cells relative to the 0 h control revealed a roughly similar fraction of unrepaired CPDs in late-replicating DNA relative to other replication categories (Figure 7c and Supplemental Figure S8c). These results are similar to our previous analysis of CPD formation in WT cells (see Figure 3). In summary, these findings indicate that in *rad16*∆ cells, late-replicating DNA has similar levels of initial CPD formation and repair as early or middle-replicating regions.

Previous studies have suggested that elevated spontaneous mutation rates in late-replicating regions might be due to increased usage of TLS polymerases to bypass lesions, as opposed to error-free mechanisms [13,25]. We wondered whether this mechanism might contribute to elevated mutation counts in late-replicating regions in *rad16*∆ cells. DNA polymerase zeta, which is required for TLS activity in yeast, often generates complex mutations, which are defined as multiple closely spaced substitutions and/or insertion/deletion (indel) events [35,36]. Hence, we identified putative complex mutation events, which we defined as two or more substitutions and/or indels each occurring within 10 bp of each other (see Materials and Methods) in individual sequenced isolates of the UVC-irradiated *rad16*∆ strain. Using these criteria, we identified a total of 522 mutations in UVC *rad16*∆ cells that were part of a complex mutation event. We then analyzed the distribution of these complex mutation events in different replication timing categories during S-phase to see if this signature of TLS activity was elevated in late-replicating regions in *rad16*∆ cells.

**Figure 8.**
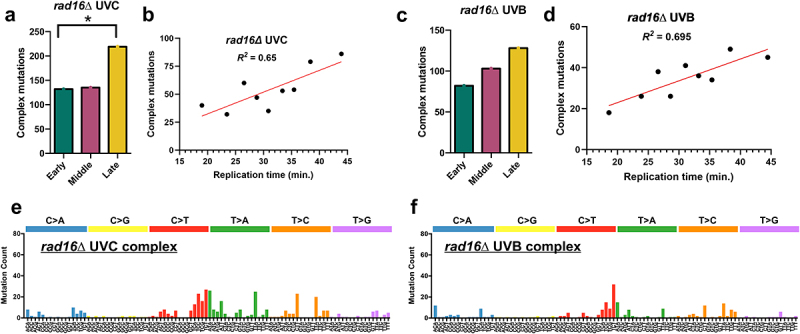
Complex mutations in UV-radiated *rad16*∆ cells are enriched in late-replicating regions. (a) The number of complex mutations in early, middle, and late replication for UVC-treated *rad16*Δ yeast. Complex mutations were defined as multiple independent substitutions and/or an indels within 10 base pairs of an adjacent mutation in the same sequenced yeast isolate. mutation data is from whole-genome sequencing of UVC-exposed yeast cells of *rad16*∆ yeast exposed to 15 doses of UVC light (12.5 J/m^2^ per dose), as described in [26]. *p < 0.05 relative to total base substitutions based on Chi-squared analysis. (b) Linear regression results plotting number of complex mutations by replication time for UVC-exposed *rad16*Δ yeast. The regression equation is y = 1.947× − 6.497 with a Pearson’s R of 0.80 and a p-value of 0.0091. Simple linear regression was performed using GraphPad prism version 10.0.3. (c) the number of complex mutations in early, middle, and late replication for UVB-treated *rad16*Δ yeast. Mutation data is from whole-genome sequencing of UVB-exposed yeast cells of *rad16*∆ yeast exposed to 15 doses of UVB light (150 J/m^2^ per dose), as described in [27]. (d) Linear regression results plotting number of complex mutations by replication time for UVB-exposed *rad16Δ* yeast. The regression equation is y = 1.063× + 1.689 with a Pearson’s R of 0.83 and *p* = 0.0052. (e) Mutation spectrum for complex single-base substitutions (C>a, C>G, C>T, T>A, T>C, or T>G) in *rad16Δ* UVC-exposed yeast cells. Counts for all trinucleotide contexts are included, with the mutated base in the middle. (f) Mutation spectrum for complex single-base substitutions in *rad16Δ* UVB-exposed yeast cells.

This analysis revealed that in UVC-irradiated *rad16*∆ cells, complex mutations are enriched in late-replicating regions of the yeast genome (Figure 8a). There were 66% and 62% more complex mutations in late-replicating regions than early or middle-replicating regions, respectively. Moreover, the distribution of complex mutations in different replication timing categories significantly differed from the expected mutation count based on the observed number of SBS in UVC-irradiated *rad16*∆ cells in each replication timing category (*p* < 0.0005). Linear regression analysis indicated that complex mutation count showed a significant positive correlation with replication timing in S-phase (Figure 8b, Pearson’s *R* = 0.80, *p* = 0.0091).

Similar analysis of putative complex mutations in UVB-irradiated *rad16*∆ cells again revealed an elevated frequency in late-replicating regions (Figure 8c). The distribution of complex mutations in different replication timing categories significantly differed from the expected counts based on sequence composition (*p* < 0.005) but did not significantly differ from expected mutation frequencies derived from the observed single-base substitution counts in UVB-irradiated *rad16*∆ cells (*p* > 0.05). Linear regression analysis confirmed that complex mutations showed a significant positive correlation with replication timing (Figure 8d, Pearson’s *R* = 0.83, *p* = 0.0052). Taken together, these findings indicate that putative complex mutations, which are a signature of TLS activity by DNA polymerase zeta, are elevated in late-replicating regions in UV-irradiated *rad16*∆ cells.

Analysis of the spectra of single-base substitutions occurring in putative complex mutations in UV-irradiated *rad16*∆ cells revealed distinct patterns of mutations (Figure 8e,f). For example, the relative proportions of C>T and T>C substitutions in dipyrimidine sequences were reduced in the complex mutation spectra (Supplemental Figure S9a) relative to the spectra of other, non-complex single-base substitutions (Supplemental Figure S9b), while the proportion of C>A, C>G, T>A, and T>G substitutions was higher in the complex mutation spectrum (Supplemental Figure S9a). These latter substitutions might reflect ‘collateral damage’ in which the TLS polymerase makes additional errors after bypassing a UV lesion [34]. Correlation analysis was performed to quantify these differences in mutation spectra. While the correlation between the *rad16*∆ complex and noncomplex mutation spectra was high (Pearson’s R 0.78 (UVC) and 0.72 (UVB), *p* < 0.0005; see Supplemental Figure S9c,d), it was reduced relative to the correlation between different replication timing categories for all single-base substitutions (Pearson’s R ~ 0.99).

We compared the mutation spectra of single-base substitutions in complex mutations in the UVC- and UVB-irradiated *rad16*∆ cells with COSMIC single base substitution signatures derived from sequenced tumors [30]. None of the COSMIC signatures were highly similar (i.e., cosine similarity >0.75) to either the *rad16*∆ UVC or UVB complex mutations (Supplemental Figure S10). However, a few COSMIC signatures showed a marginal cosine similarity (i.e., cosine similarity >0.59), with COSMIC signatures SBS7a, SBS40a, and SBS40c showing marginal similarity to both the *rad16*∆ UVC and UVB complex mutations (Supplemental Figure S10). SBS7a is highly enriched in skin cancers such as melanoma, and is known to be caused by UV exposure. SBS40a and SBS40c have an unknown etiology, but are found in many cancer types [37]. Taken together, these findings suggest a model in which elevated TLS activity in late-replicating regions promotes UV mutagenesis in GG-NER deficient *rad16*∆ cells.

## Discussion

Previous studies have indicated that the timing in which a genomic region is replicated during S-phase significantly impacts the frequency of mutations in many cancer types, including in skin cancers [13–15], as well as the frequency of spontaneous mutations in yeast [9–12]. However, the mechanism responsible for this effect remains poorly understood, with a number of competing hypotheses proposed. Here, we explored the impact of replication timing on UV mutagenesis in yeast, using a compendium of >60,000 UV-induced mutations derived from repair-proficient and repair-deficient strains. This analysis indicated that in GG-NER proficient yeast, UV-induced single-base substitutions are significantly elevated in early-replicating regions of the genome. In contrast, in GG-NER deficient *rad16*∆ cells, UV-induced mutations are elevated in late-replicating regions. Investigation of genome-wide maps of CPD formation and repair indicate that UV-induced CPD formation is specifically elevated in late-replicating regions of the genome, but this was determined to be specifically due to CPD formation in late-replicating ribosomal DNA (rDNA) repeats. Our analysis indicates that complex mutations, which are a signature of TLS polymerase activity, are also elevated in late-replicating regions in *rad16*∆ cells. Taken together, these findings suggest that elevated TLS activity contributes to elevated mutation rates in late-replicating regions in *rad16*∆ cells, while GG-NER activity in WT cells suppresses mutations in late-replicating regions.

Surprisingly, WT yeast exposed to UVC and UVB irradiation show a consistently elevated frequency of mutations in early-replicating regions of the genome. The magnitude of this increase was relatively small, ranging from 8% to 13% higher than the mutation count in late-replicating regions; however, this effect size is roughly comparable to the ~20–30% increase in mutation frequency in late-replicating regions observed in previous genome-wide studies analyzing spontaneous mutations or SNPs in yeast [14,23]. This finding is unexpected, since previous studies of spontaneous mutations in yeast and somatic mutations in human cancers generally saw the opposite trend, with fewer mutations in early-replicating regions relative to late-replicating DNA. However, at least two mutational processes operating in cancer cells are enriched in early-replicating DNA, namely mutations arising from APOBEC cytidine deaminases and a subset of UV-induced mutations in melanoma associated with COSMIC signature 7b [21,22]. This latter finding is consistent with the UV-induced mutations we observed in repair-proficient yeast, which are also enriched in early-replicating DNA. In contrast, UV-induced somatic mutations in melanoma associated with COSMIC signature 7a are enriched in late-replicating regions [22].

Our analysis of published CPD-seq maps [28,29] indicates that the enrichment of UV-induced mutations in early-replicating regions of the yeast genome is unlikely to be a consequence of elevated UV damage formation or repair inhibition. Indeed, our initial analysis of CPD formation revealed ~20–25% more damage in late-replicating regions of the genome relative to early or middle-replicating regions. A previous study of UV damage formation in human cells also observed increased CPD levels in late-replicating heterochromatin regions associated with the nuclear lamina [38]. However, in this prior study, the authors concluded that elevated UV damage formation was due to the positioning of late-replicating regions near the nuclear periphery [38], while our analysis of CPD-seq data in yeast indicated that elevated CPD formation in late-replicating regions of the yeast genome is entirely due to repetitive rDNA sequences. Once the repetitive rDNA was excluded, roughly similar levels of CPD formation were observed in early and late-replicating regions of the genome. In the future, it will be intriguing to investigate the impact of replication timing on more rare forms of UV damage, such as pyrimidine-pyrimidone [4] photoproducts and atypical thymine-adenine (TA) photoproducts [39,40]. Our analysis of repair of CPD lesions across the genome indicates that replication timing in yeast also does not significantly modulate repair efficiency. In contrast, late replicating regions in human cells tend to be heterochromatic and therefore less accessible to repair factors, which is thought to be a primary cause of elevated somatic mutation rates in late-replicating regions of the human genome [16,19,41,42]. In summary, our analysis of genome-wide CPD-seq data indicates that postulated differences in repair efficiency or damage formation cannot explain the enrichment of UV-induced mutations in early-replicating regions of the genome in WT yeast.

**Figure 9.**
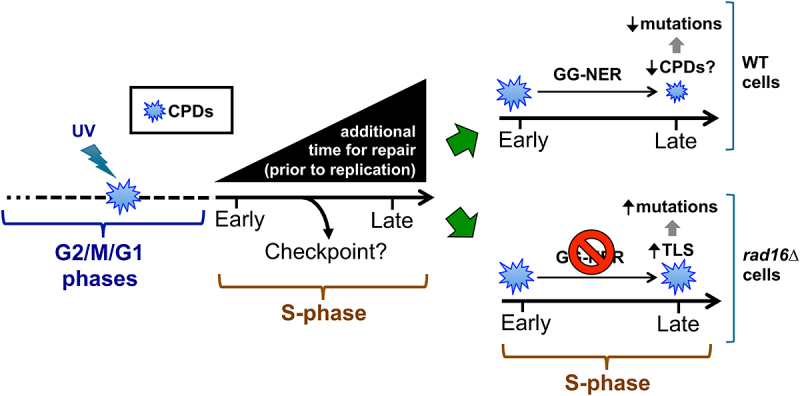
Model of how replication timing affects mutation frequency by regulating the amount of time available to repair CPD lesions formed during the G2, M, or G1 (i.e., G2/M/G1) phases of the cell cycle prior to replication. We propose that increased time to repair during S-phase suppresses UV mutation rates in late-replicating regions in GG-NER proficient cells, while elevated TLS activity in late-replicating regions promotes UV mutagenesis in GG-NER deficient cells.

Instead, we propose a model in which mutations accumulate in early-replicating regions because there is less time for the NER machinery to repair lesions in such regions prior to mutations being fixed by replication (Figure 9). Previous studies indicate that S-phase in undamaged yeast cells requires ~25 min to complete [43]. Hence, for yeast cells that were UV-irradiated in the G1, M, or G2 phases of the cell cycle, there could be as much as a 25-min difference in the time available to repair for damage located in an early-replicating region of the genome relative to a late-replicating region. Since the yeast cell cycle as a whole requires only ~90–120 min to complete, this extra time likely results in lower numbers of unrepaired CPDs in late-replicating regions prior to replication (Figure 9). This difference in repair time between early and late-replicating regions of the genome would be even greater if such cells enter the intra-S-phase checkpoint after initiating replication, due to replication stress arising from the presence of unrepaired UV damage [44]. In contrast, in cells irradiated in the G1, M, or G2 phases of the cell cycle, early-replicating regions should have the least amount of time to repair DNA lesions prior to replication (Figure 9), and this can potentially explain our observation that UV-induced mutations are elevated in early-replicating regions of the genome.

In support of this model, deletion of *RAD16*, which is required for GG-NER in yeast [45], eliminates the enrichment of UV-induced mutations in early-replicating regions. Instead, UV-irradiated *rad16*∆ cells show a significant enrichment of single-base substitutions in late-replicating regions, with >30% more mutations than in early-replicating regions of the genome. Our analysis of genome-wide CPD-seq data in yeast indicates that UV-induced CPD formation is roughly similar in each replication timing category, once the repetitive rDNA locus is excluded. Similar analysis of genome-wide repair data from *rad16*∆ cells (presumably reflecting ongoing TC-NER) suggests that repair of CPDs is also roughly equivalent in genomic regions with different replication timing. While our analysis suggests that elimination of GG-NER in yeast enhances mutation frequency in late-replicating regions, previous studies in human skin cancers indicate that loss of GG-NER (*e.g.*, in *XPC*^*-/-*^ skin cancers) reduces the enrichment of mutations in late-replicating regions [16,17], likely because late-replicating regions in human cells are largely heterochromatic and, therefore, normally inaccessible to the GG-NER machinery. Hence, this discrepancy can be at least in part explained by differences in chromatin organization and repair accessibility between early- and late-replicating regions in yeast and human cells.

In yeast, the expression of *REV1*, which is essential for TLS activity by DNA polymerase zeta (pol zeta), is restricted to late S and G2/M phases of the cell cycle [25]. A previous study suggested that this temporal regulation of TLS activity promotes elevated rates of spontaneous mutations in late-replicating regions of yeast chromosome VI [13]. Consistent with this hypothesis, analysis of the replication timing of putative complex mutation events, which are a signature of pol zeta TLS activity [35,36], revealed that in UV-irradiated *rad16*∆ cells, such mutation classes are enriched in late-replicating regions. Hence, elevated TLS activity in late-replicating genomic regions may promote higher mutation rates in these regions in *rad16*∆ cells (Figure 9). In contrast, no analogous temporal regulation has been demonstrated for human REV1. Differential expression of the mutagenic *REV1* TLS polymerase throughout S-phase in yeast could contribute to the effect of replication timing on mutation frequency, especially in GG-NER deficient cells, and may account for the discrepancy between the impact of replication timing on UV mutagenesis in GG-NER deficient yeast cells (i.e., *rad16*∆) and human tumors (i.e., *XPC*^*-/-*^).

Our preliminary analysis indicates that complex mutations are also enriched in late-replicating regions in UVC-irradiated WT cells (Supplementary Figure S11), consistent with our analysis of UVC-exposed *rad16*∆ cells. However, preliminary analysis of complex mutations in WT cells irradiated with UVB light did not show enrichment in late-replicating regions, but instead were enriched in early-replicating regions (Supplementary Figure S11). While UVB-irradiated *rad16*∆ cells showed elevated complex mutations in late replicating regions (Figure 8c), the observed enrichment was less than that observed for UVC-irradiated *rad16*∆ cells, and not statistically significant when compared to the expected number of complex mutations based on the observed patterns of single-base substitutions. It is possible that wavelength-dependent differences in UV photoproduct formation may in part explain this discrepancy. For instance, UVC exposure is thought to yield a higher proportion of 6-4PPs and atypical thymine-adenine (TA) photoproducts than UVB treatment [46,47], and mutagenic bypass of both of these photoproducts is thought to involve pol zeta [48–50]. We hypothesize that mutagenic bypass of these helix-distorting photoproducts by pol zeta may be responsible for the enrichment of complex mutations in late-replicating regions in UVC-irradiated cells.

In conclusion, these findings indicate that the interplay of replication timing, repair activity, and translesion DNA synthesis shapes the frequency of UV-induced mutations during S-phase. Since replication timing also significantly impacts UV mutagenesis in human skin cancers, these findings have potentially important implications for understanding the origin of mutational heterogeneity in human cancer genomes.

## Supplementary Material

Supplemental Material

## Data Availability

The data that support the findings of this study are available in NCBI Sequence Read Archive (SRA; https://www.ncbi.nlm.nih.gov/sra) under BioProject accession number SRA: PRJNA605561 (https://www.ncbi.nlm.nih.gov/bioproject/?term=(PRJNA605561)) and PRJNA888347 (https://www.ncbi.nlm.nih.gov/bioproject/?term=(PRJNA888347)), and from the Gene Expression Omnibus (GEO) database (https://www.ncbi.nlm.nih.gov/geo/) under accessions: GSE79977 (https://www.ncbi.nlm.nih.gov/geo/query/acc.cgi?acc=GSE79977) and GSE131101 (https://www.ncbi.nlm.nih.gov/geo/query/acc.cgi?acc=GSE131101). Software code is available on Zenodo at https://zenodo.org/records/14990403. Source data for graphs are included in Supplemental Data File 1 and is available on Zenodo at https://zenodo.org/records/15009048. The authors confirm that the data supporting the findings of this study are available in the article and its supplemental materials.
