# Supplementary material for: Interplay of replication timing, DNA repair, and translesion synthesis in UV mutagenesis in yeast
Source: Nucleus. 2025 Mar 13;16(1):2476935. doi: 10.1080/19491034.2025.2476935 (PMC11913381; doi:10.1080/19491034.2025.2476935)
Supplement: Supplemental Material [file KNCL_A_2476935_SM9779.docx]

**Supplemental Materials**

**Supplemental Figure Legends**


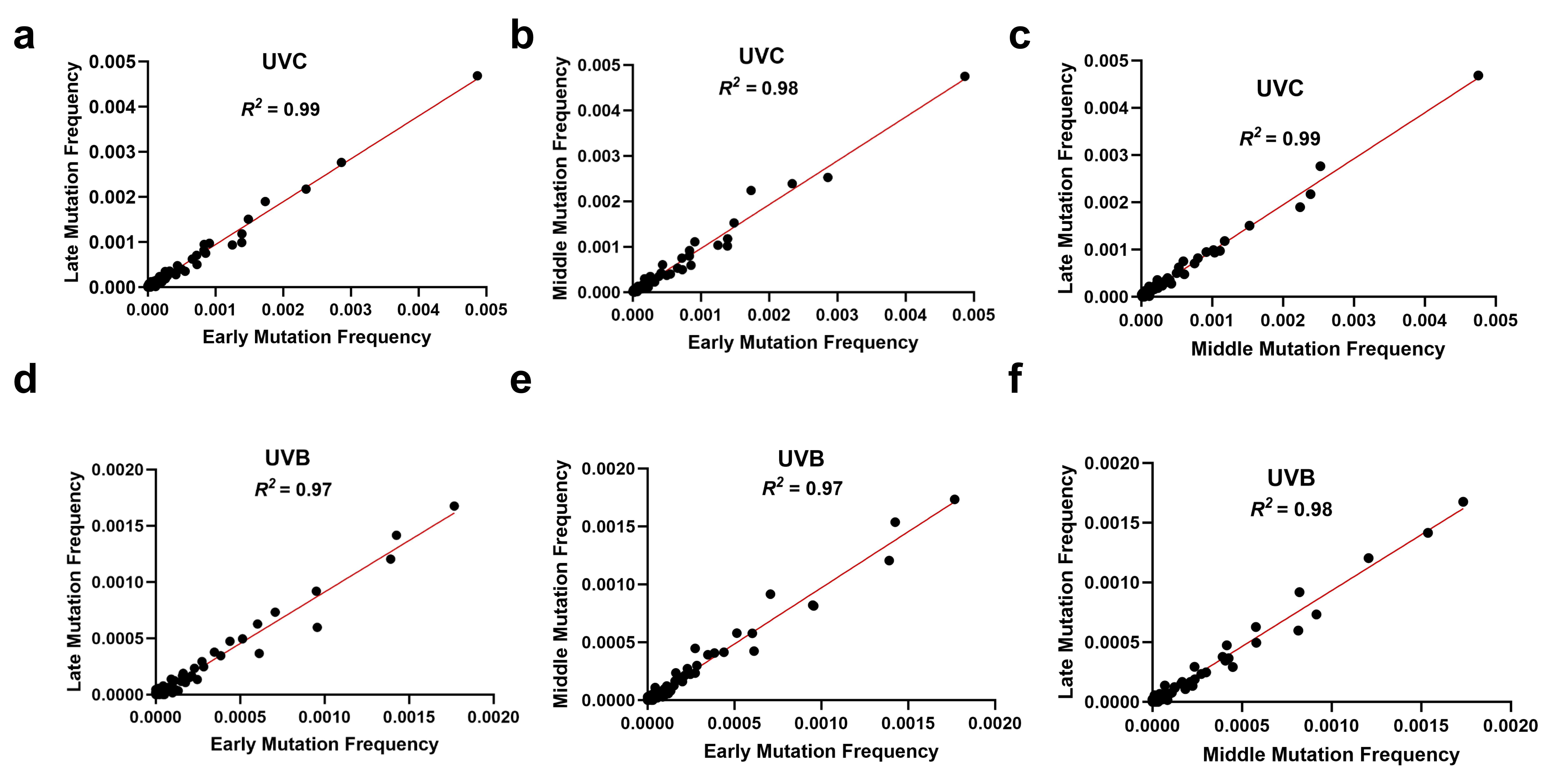


**Supplemental Figure 1**. Comparison of mutation spectra associated with genomic regions with different replication timing in UV-radiated WT yeast. Mutation frequencies for each trinucleotide context were compared for different replication times for wild-type yeast cells exposed to UVC (a-c) or UVB (d-f) as described in (1, 2). Linear regression analysis was performed using GraphPad Prism version 10.0.3.​ (a) Correlation between mutations in early and late replication. P < 0.0005.​ (b) Correlation between mutations in early and middle replication. P < 0.0005.​ (c) Correlation between mutations in middle and late replication. P < 0.0005. (d) Correlation between mutations in early and late replication. P < 0.0005.​ (e) Correlation between mutations in early and middle replication. P < 0.0005.​ (f) Correlation between mutations in middle and late replication. P < 0.0005.


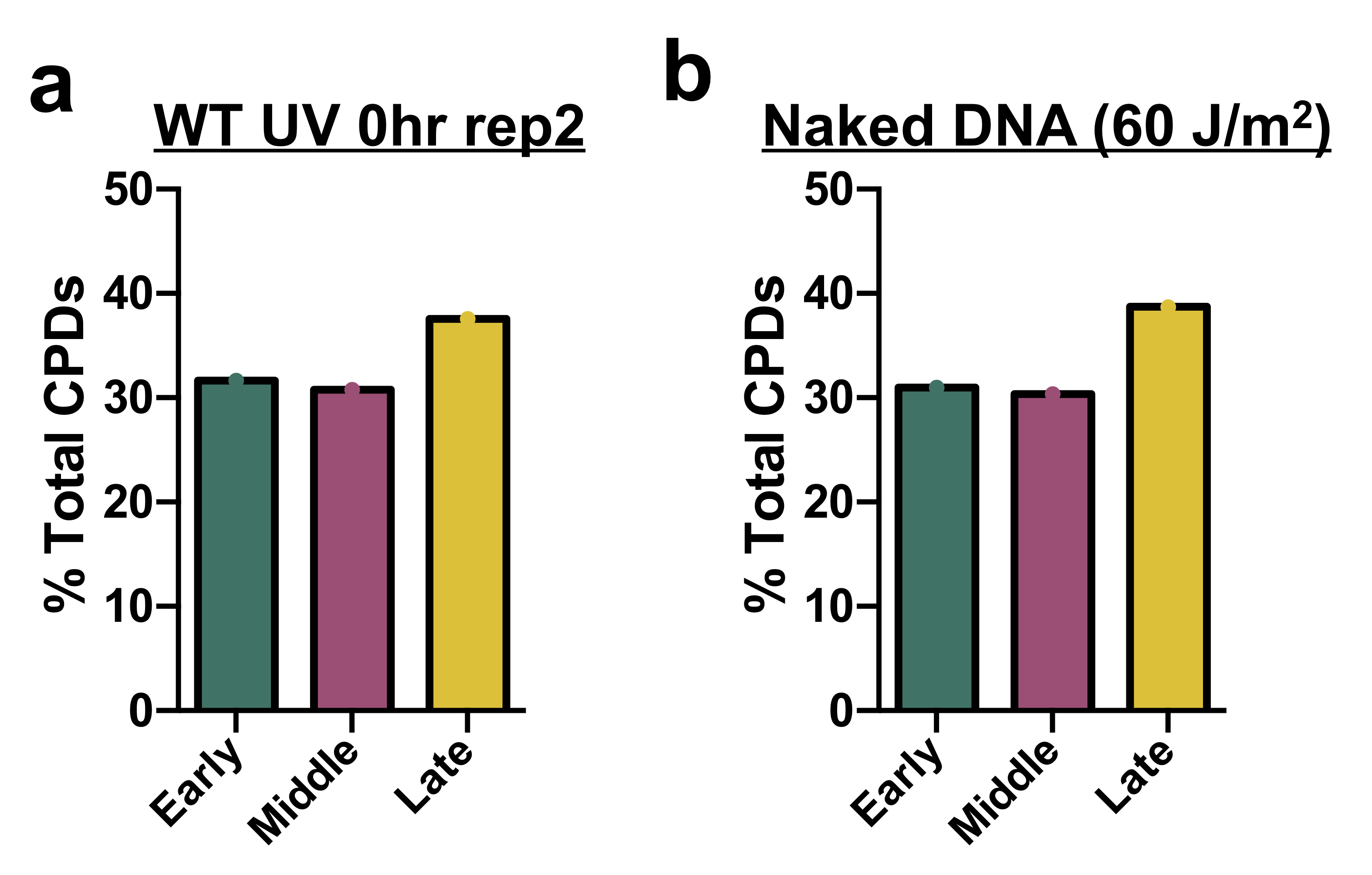


**Supplemental Figure 2**. Analysis of UV-induced CPD formation and repair in early, middle, and late replicating regions of the yeast genome. (a) Percentage of CPDs in early, middle, and late-replicating regions of the yeast genome immediately after UVC-radiation of WT cells (0hr – replicate 2). CPD counts determined from published CPD-seq data (3). (b) Same as panel a, except for UVC-radiation (60 J/m^2^) of isolated yeast genomic DNA.


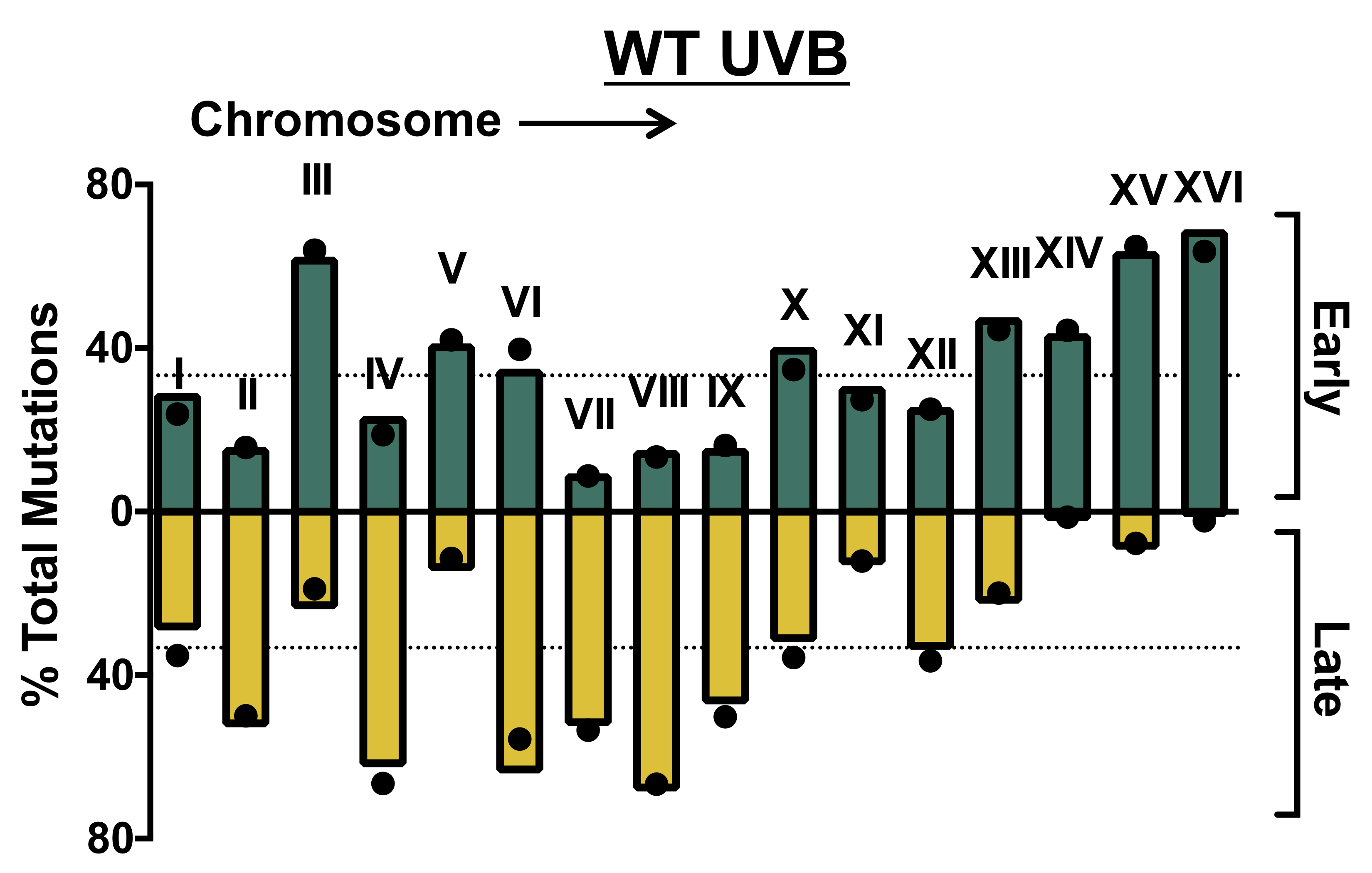


**Supplemental Figure 3**. Mutation percentages in early- and late-replicating DNA (green and yellow bars, respectively), derived from whole-genome sequencing of UVB-exposed yeast cells (1) and plotted for each yeast chromosome. Expected values (black dots) were calculated based on the DNA sequence context using trinucleotide counts by region (early, middle, or late-replicating) and overall mutation frequencies for each trinucleotide context. Individual chromosomes are labeled. The dotted lines represent the expected proportion of mutations (i.e., 33% or 1/3^rd^) for early and late-replicating regions across the genome as a whole.


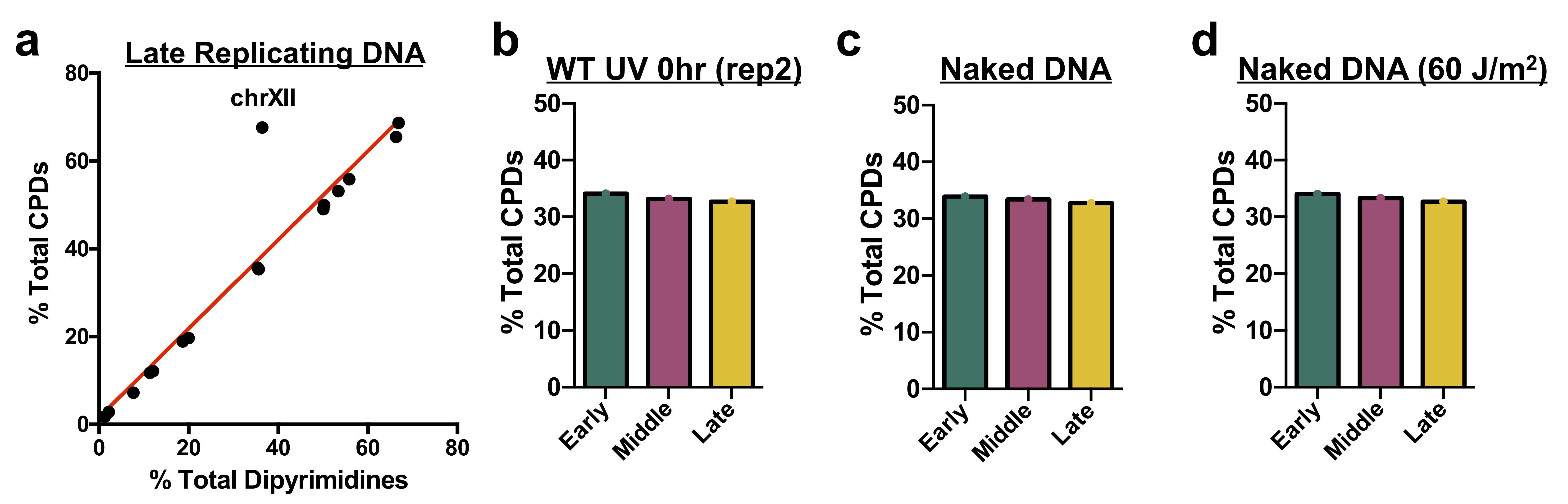


**Supplemental Figure 4**. Analysis of UV-induced CPD formation and repair in early, middle, and late replicating regions of the yeast genome. (a) Plot of percentage of total CPDs in late replicating DNA for each chromosome relative to the percentage of dipyrimidine sequences in late replicating DNA. Each point represents a single chromosome. Chromosome (chr) XII is labeled. CPD-seq data is of UV-radiated WT cells, 0hr (3). (b) Percentage of CPDs in early, middle, and late-replicating regions of the yeast genome immediately after UVC-radiation of WT cells (0hr – replicate 2) after removing CPDs associated with ribosomal DNA (rDNA) repeats. CPD counts determined from published CPD-seq data (3). (c-d) Same as panel b, except for UVC-radiation of either (c) 90 J/m^2^ or (d) 60 J/m^2^ of isolated yeast genomic DNA after removing CPDs associated with ribosomal DNA (rDNA) repeats.


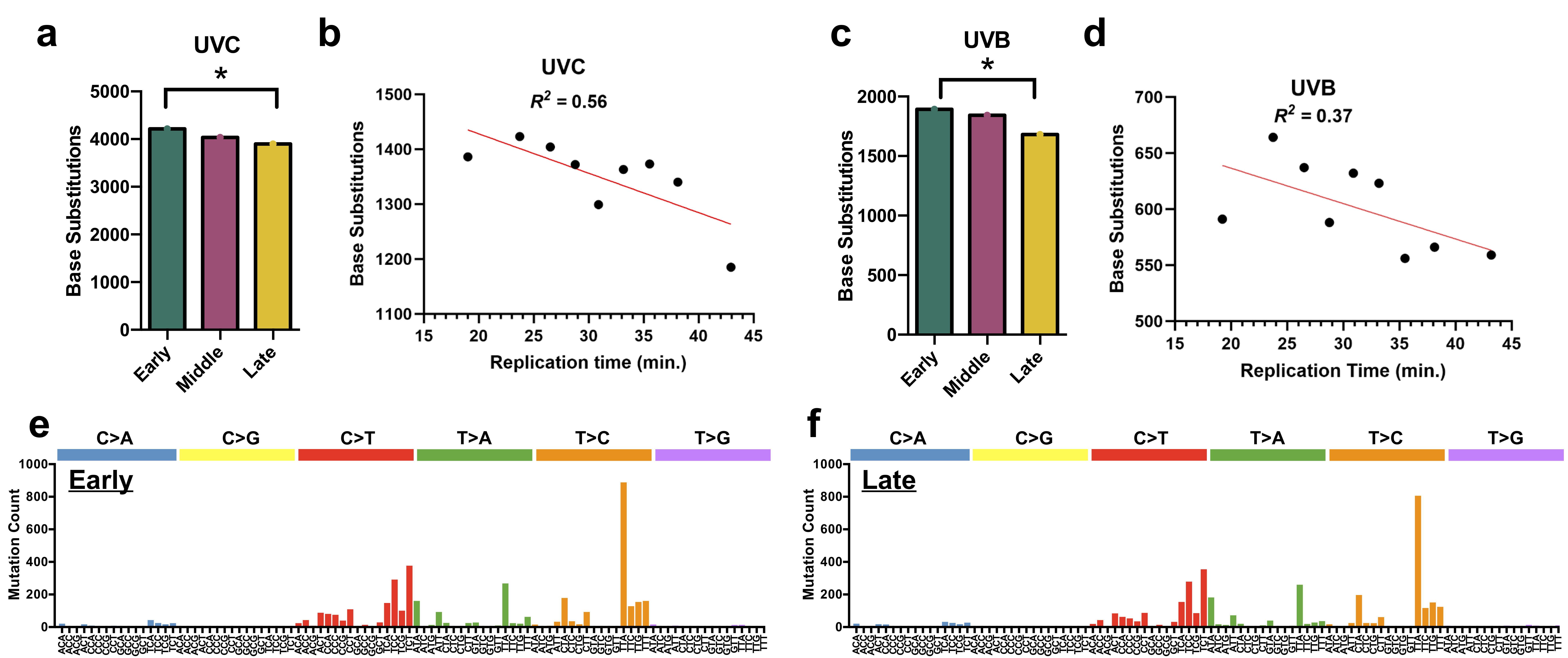


**Supplemental Figure 5.** UV-induced single base substitutions in wild-type (WT) cells are still elevated in early-replicating regions of the yeast genome when rDNA regions on chromosome XII are filtered out. (a) The number of single-base substitutions in early, middle, and late-replicating regions of the genome for UVC-treated wild-type (WT) yeast. Mutations are derived from genome-wide sequencing of WT yeast exposed to 15 doses of UVC light (25 J/m^2^ per dose), as described in (26). *P < 0.05 based on chi-square analysis of observed mutation counts relative to the expected mutations counts based on tri-nucleotide sequence contexts of the early, middle, and late-replicating genomic sequences. (b) Linear regression results plotting number of single-base substitution mutations by replication time for UVC-exposed WT yeast. The regression equation is y = -7.173x + 1572  with a Pearson’s R of -0.75 and a  p-value of 0.0209. Simple linear regression was performed using GraphPad Prism version 10.0.3. (c) The number of single-base substitutions in early, middle, and late-replicating regions of the genome for UVB-treated wild-type (WT) yeast. Mutations are derived from genome-wide sequencing of WT yeast exposed to 15 doses of UVB light (300 J/m^2^ per dose), as described in (27). *P < 0.05 based on chi-square analysis. (d) Linear regression results plotting number of single-base substitution mutations by replication time for UVB-exposed wild-type yeast. The regression equation is y = -3.158x + 699.7 with a Pearson’s R of -0.61 and a p-value of 0.0841. (e ) Mutation spectrum for single-base substitutions (C>A, C>G, C>T, T>A, T>C, or T>G) in early S-phase for WT UVC-exposed yeast cells. Counts for all trinucleotide contexts are included, with the mutated base in the middle. (f) Mutation spectrum for single-base substitutions in late S-phase for WT UVC-exposed yeast cells.


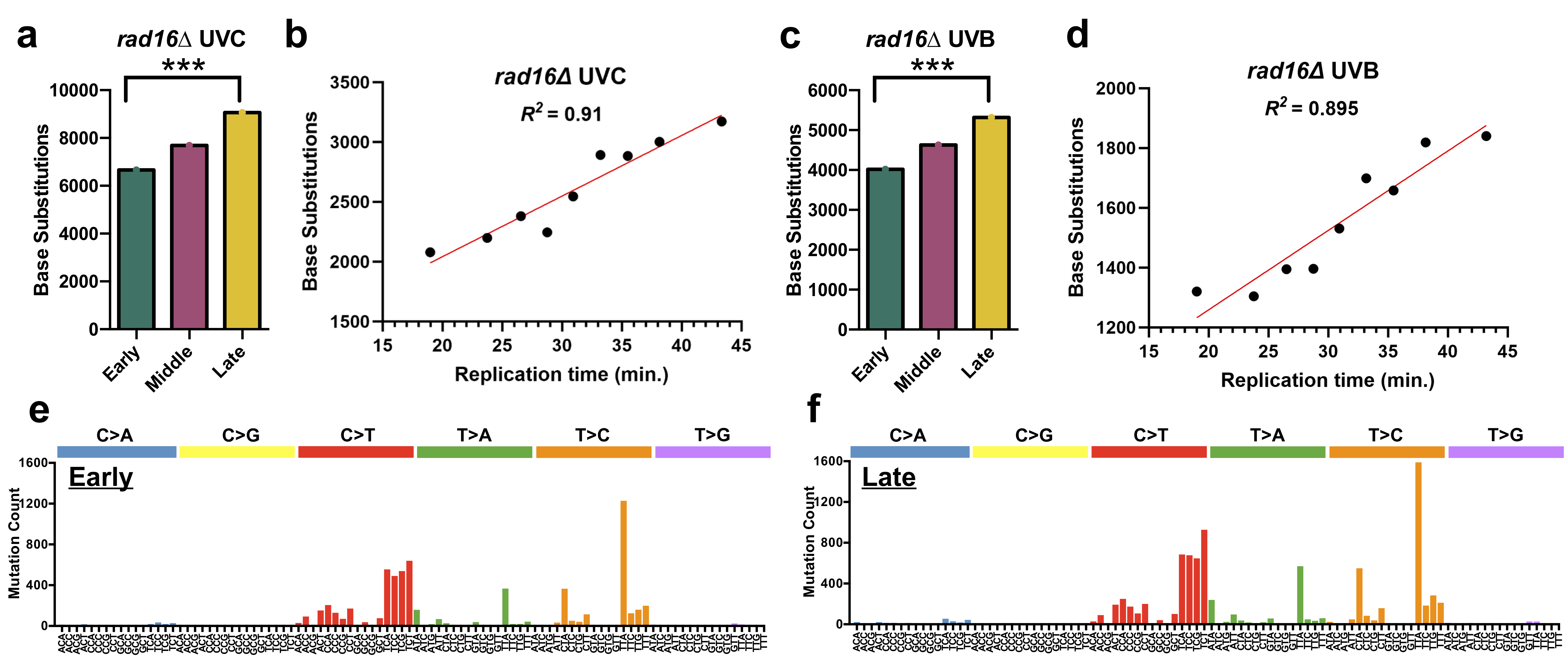


**Supplemental Figure 6**. UV-induced single base substitutions in *rad16*∆ cells are still elevated in late-replicating regions of the yeast genome when rDNA regions on chromosome XII are filtered out. (a) The number of single-base substitutions in early, middle, and late-replicating regions of the genome for UVC-treated *rad16*∆ yeast. Mutations are derived from genome-wide sequencing of *rad16*∆ yeast exposed to 15 doses of UVC light (12.5 J/m^2^ per dose), as described in (26). ***P < 0.0005 based on chi-square analysis of observed mutation counts relative to the expected mutations counts based on tri-nucleotide sequence context of the early, middle, and late-replicating genomic sequences. (b)  Linear regression results plotting number of single-base substitution mutations by replication time for UVC-exposed *rad16*Δ yeast. The regression equation is y = 50.67x + 1029 with a Pearson’s R of 0.96 and a p-value of < 0.0005. Simple linear regression was performed using GraphPad Prism version 10.0.3. (c) The number of single-base substitutions in early, middle, and late-replicating regions of the genome for UVB-treated *rad16*∆ yeast. Mutations are derived from genome-wide sequencing of WT yeast exposed to 15 doses of UVB light (150 J/m^2^ per dose), as described in (27). ***P < 0.0005 based on chi-square analysis. (d) Linear regression results plotting number of single-base substitution mutations by replication time for UVB-exposed *rad16Δ* yeast. The regression equation is y = 26.57x + 727.7, with a Pearson’s R of 0.95 and a  p-value < 0.0005. (e) Mutation spectrum for single-base substitutions (C>A, C>G, C>T, T>A, T>C, or T>G) in early S-phase for *rad16*∆ UVC-exposed yeast cells. Counts for all trinucleotide contexts are included, with the mutated base in the middle. (f) Mutation spectrum for single-base substitutions in late S-phase for *rad16*∆ UVC-exposed yeast cells.


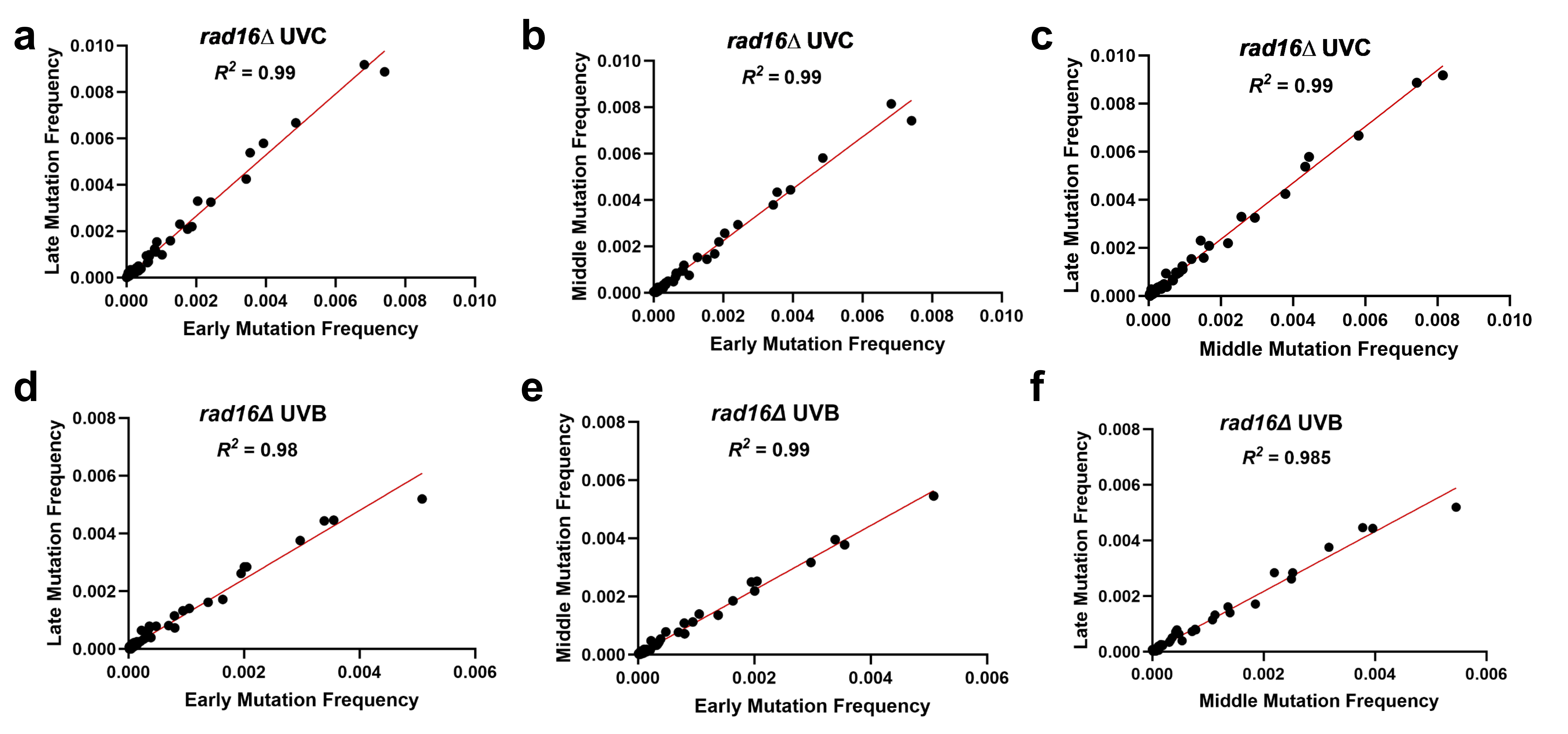


**Supplemental Figure 7**. Comparison of mutation spectra associated with genomic regions with different replication timing in UV-radiated *rad16*∆ yeast. Mutation frequencies for each trinucleotide context at different replication times for *rad16*∆ yeast cells exposed to UVC (a-c) or UVB (d-f). Data from (1, 2). Correlation/linear regression analysis was performed using GraphPad Prism version 10.0.3. (a) Correlation between mutations in early and late replication timing for *rad16*∆ UVC single base substitutions. P < 0.0005.​ (b) Correlation between mutations in early and middle replication timing for *rad16*∆ UVC single base substitutions. P < 0.0005.​ (c) Correlation between mutations in middle and late replication timing for *rad16*∆ UVC single base substitutions. P < 0.0005. (d) Correlation between mutations in early and late replication timing for *rad16*∆ UVB single base substitutions. P < 0.0005.​ (e) Correlation between mutations in early and middle replication timing for *rad16*∆ UVB single base substitutions. P < 0.0005.​ (f) Correlation between mutations in middle and late replication timing for *rad16*∆ UVB single base substitutions. P < 0.0005.


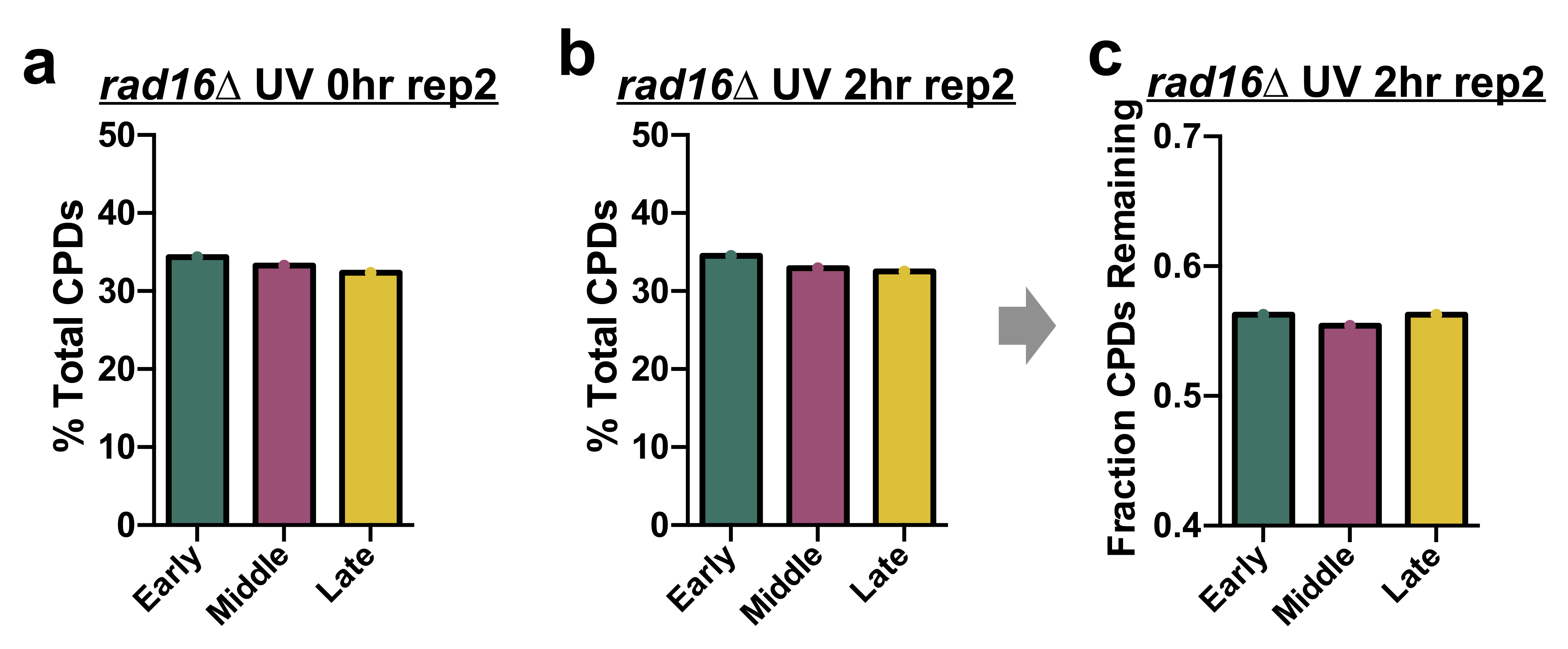


**Supplemental Figure 8**. Analysis of UV-induced CPD formation and repair in early, middle, and late replicating regions of the yeast genome in *rad16*∆ cells. (a) Percentage of CPDs in early, middle, and late-replicating regions of the yeast genome immediately after UVC-radiation of *rad16*∆ cells (0hr – replicate 2). CPD counts determined from published CPD-seq data (4) after removing CPDs associated with ribosomal DNA (rDNA) repeats. (b) Same as panel a, except after 2hr of repair in UV-radiated *rad16*∆ cells (2hr). (c) Fraction of CPDs remaining after 2hr of repair in UV-radiated *rad16*∆ cells relative to the 0hr control in early, middle, and late-replicating regions of the yeast genome, after removing CPDs associated with ribosomal DNA (rDNA) repeats.


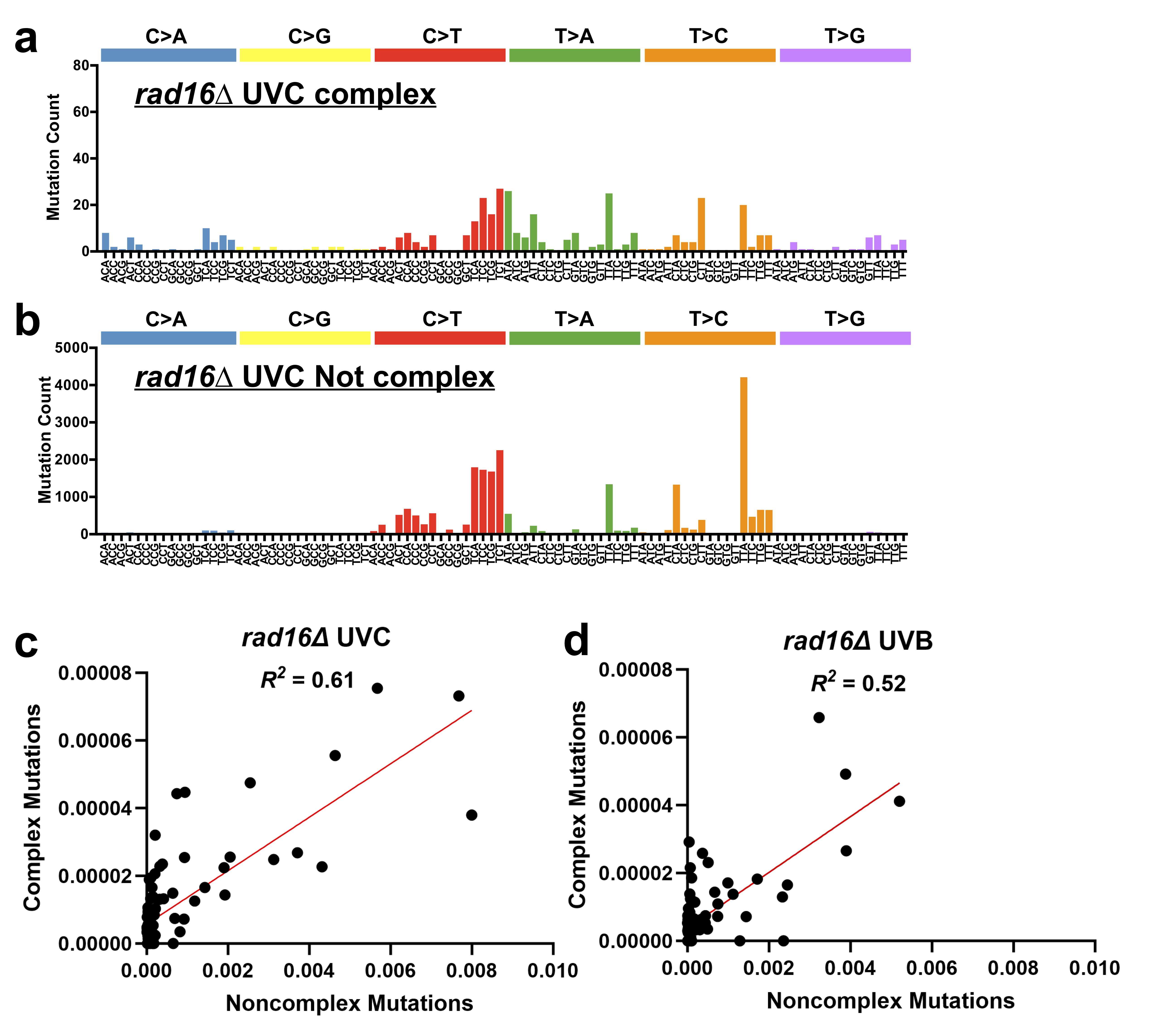


**Supplemental Figure 9**. (a-b) Comparison of mutation spectra for each trinucleotide context for complex and noncomplex mutations in *rad16*∆ yeast cells exposed to UVC. Complex mutations were defined as multiple independent substitutions and/or an indels within 10 base pairs of an adjacent mutation in the same sequenced yeast isolate. Panel a is the same as figure 8e. ​(c-d) Correlation/linear regression analysis of frequencies of mutations in different trinucleotide contexts for complex and noncomplex mutations in *rad16*∆ yeast cells to (c) UVC or (d) UVB. Correlation/linear regression analysis was performed using GraphPad Prism version 10.0.3, with (c) Pearson’s R = 0.78 and P < 0.0005 and (d) Pearson’s R = 0.72 and P <0.0005.


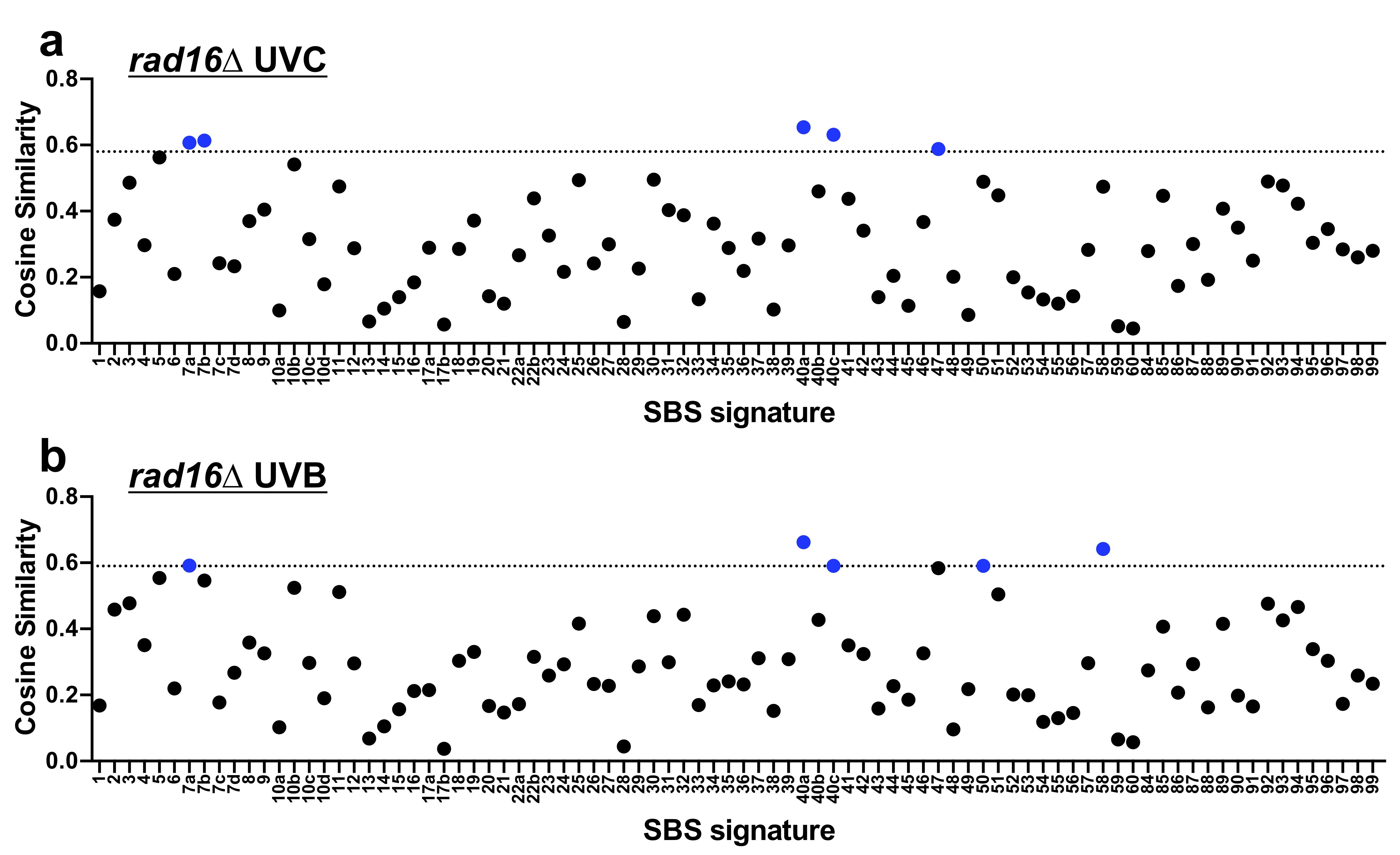


**Supplemental Figure 10**. Comparison of mutation spectra for the set of *rad16*∆ SBS complex mutations in yeast cells exposed to (a) UVC and (b) UVB and the mutation signatures extracted from the COSMIC database. Cosine similarities were determined by treating the lists of mutation frequencies as vectors and dividing the dot product by the product of the vector magnitudes. Cosine similarities greater than 0.59 are indicated in blue.


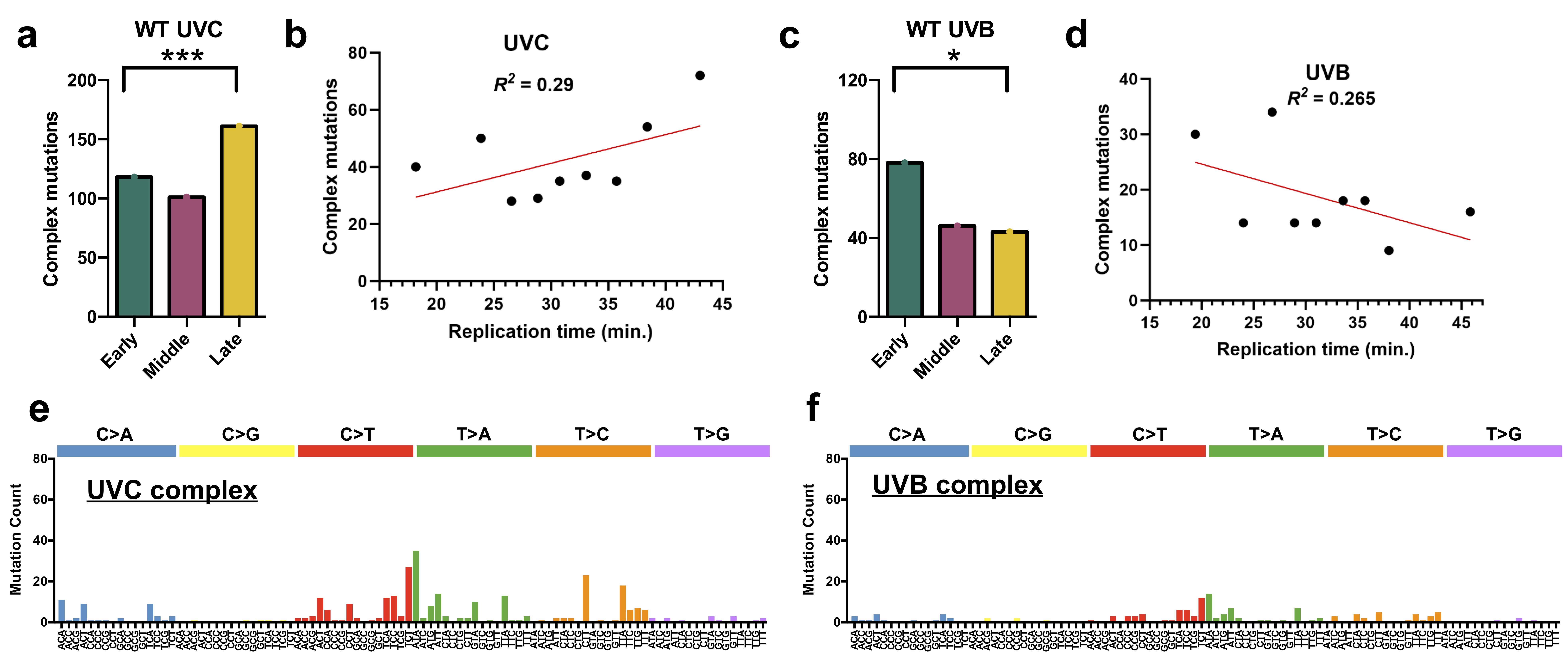


**Supplemental Figure 11**. Complex mutations in UV-irradiated WT cells. (a) The number of complex mutations in early, middle, and late replication for UVC-treated WT yeast. Complex mutations were defined as multiple independent substitutions and/or an indels within 10 base pairs of an adjacent mutation in the same sequenced yeast isolate. ​Mutation data is from whole-genome sequencing of UVC-exposed yeast cells of WT yeast exposed to 15 doses of UVC light (25 J/m^2^ per dose), as described in (26). ***P < 0.0005 relative to total base substitutions based on Chi-squared analysis.​ (b) Linear regression results plotting number of complex mutations by replication time for UVC-exposed WT yeast. The regression equation is y = 1.003x + 11.2 with Pearson’s R of 0.54 and a p-value of 0.1326. Simple linear regression was performed using GraphPad Prism version 10.0.3. ​(c) The number of complex mutations in early, middle, and late replication for UVB-treated WT yeast. Mutation data is from whole-genome sequencing of UVB-exposed yeast cells of WT yeast exposed to 15 doses of UVB light (300 J/m^2^ per dose), as described in (27). *P <0.05 (d) Linear regression results plotting number of complex mutations by replication time for UVB-exposed WT yeast. The regression equation is y = -0.5223x + 36 with a Pearson’s R of -0.53 and a p-value of 0.146. (e) Mutation spectrum for complex single-base substitutions (C>A, C>G, C>T, T>A, T>C, or T>G) in WT UVC-exposed yeast cells. Counts for all trinucleotide contexts are included, with the mutated base in the middle.​​​ (f) Mutation spectrum for complex single-base substitutions in WT UVB-exposed yeast cells.

**Supplemental References**

1. Laughery MF*, et al.* (2023) Genome-wide maps of UVA and UVB mutagenesis in yeast reveal distinct causative lesions and mutational strand asymmetries. *Genetics* 224(3):iyad086.

2. Laughery MF*, et al.* (2020) Atypical UV Photoproducts Induce Non-canonical Mutation Classes Associated with Driver Mutations in Melanoma. *Cell reports* 33(7):108401.

3. Mao P, Smerdon MJ, Roberts SA, & Wyrick JJ (2016) Chromosomal landscape of UV damage formation and repair at single-nucleotide resolution. *Proceedings of the National Academy of Sciences of the United States of America* 113(32):9057-9062.

4. Mao P, Smerdon MJ, Roberts SA, & Wyrick JJ (2020) Asymmetric repair of UV damage in nucleosomes imposes a DNA strand polarity on somatic mutations in skin cancer. *Genome research* 30(1):12-21.
